# Short term exposure to cooking fumes and pulmonary function

**DOI:** 10.1186/1745-6673-4-9

**Published:** 2009-05-04

**Authors:** Sindre Svedahl, Kristin Svendsen, Torgunn Qvenild, Ann Kristin Sjaastad, Bjørn Hilt

**Affiliations:** 1 Department of Occupational Medicine, Norwegian University of Science and Technology, Trondheim, Norway; 2 Department of Cancer research and Molecular Medicine, The Faculty of Medicine, Norwegian University of Science and Technology, Trondheim, Norway; 3 Department of Industrial Economics and Technology Management, Norwegian University of Science and Technology, Trondheim, Norway; 4 Department of Occupational Medicine, St. Olavs University Hospital, Trondheim, Norway

## Abstract

**Background:**

Exposure to cooking fumes may have different deleterious effects on the respiratory system. The aim of this study was to look at possible effects from inhalation of cooking fumes on pulmonary function.

**Methods:**

Two groups of 12 healthy volunteers (A and B) stayed in a model kitchen for two and four hours respectively, and were monitored with spirometry four times during twenty four hours, on one occasion without any exposure, and on another with exposure to controlled levels of cooking fumes.

**Results:**

The change in spirometric values during the day with exposure to cooking fumes, were not statistically significantly different from the changes during the day without exposure, with the exception of forced expiratory time (FET). The change in FET from entering the kitchen until six hours later, was significantly prolonged between the exposed and the unexposed day with a 15.7% increase on the exposed day, compared to a 3.2% decrease during the unexposed day (p-value = 0.03). The same tendency could be seen for FET measurements done immediately after the exposure and on the next morning, but this was not statistically significant.

**Conclusion:**

In our experimental setting, there seems to be minor short term spirometric effects, mainly affecting FET, from short term exposure to cooking fumes.

## Background

Exposure to cooking fumes is abundant both in domestic homes and in professional cooks and entails a possible risk of deleterious health effects. When food is cooked at temperatures up to 300°C, carbohydrates, proteins, and fat are reduced to toxic products, such as aldehydes and alkanoic acids[1-4] which can cause irritation of the airway mucosa[5-8]. Cooking fumes also contains carcinogenic and mutagenic compounds, such as polycyclic aromatic hydrocarbons and heterocyclic compounds[1-3,9-13]. Exposure to cooking fumes has also been associated in several studies with an increased risk of respiratory cancer[14-18]. Recently, the International Agency for Research on Cancer has classified emissions from high temperature frying as probably carcinogenic to humans[19].

Frying at high temperatures also produces aerosols of fat with small aerodynamic diameters of 20–500 nm which disperse in the air of the kitchen and nearby facilities. Such aerosols, containing fatty acids, irritate the airway mucosa, and can cause pneumonia[20-22]. It has also been shown that the inhalation of aerosols of oil mist from other kinds of oils can cause small airway obstruction[23-25]. Chinese investigations have shown that exposure to cooking fumes at work can be associated with rhinitis[26], respiratory disorders, and impaired pulmonary function[27]. In two Norwegian studies, it has been shown that cooks and kitchen workers had an increased occurrence of respiratory distress associated with work[28] and increased mortality from airway disease[29]. Few other studies have addressed the biological effects of exposure to cooking fumes in western domestic and professional kitchens.

Spirometry is the most common, and also a quite sensitive pulmonary function test. It has been used for a long time in many investigations, for detecting chronic work-related impaired lung function in general, but it has also been possible to study short term cross-shift changes in different settings[30,31]. The traditional spirometric time-volume curve measures the bowl function of the lungs, while flow-volume curves and other measures also give indications of the function of the smaller and more peripheral airways.

The aim of this study was to see if short term exposure to moderate levels of cooking fumes in an indoor environment causes changes in pulmonary function.

## Methods

Twenty four voluntary non-smoking students without any chronic or current respiratory disease were recruited for the study. They were split into group A which consisted of 8 males and 4 females, and group B with 7 males and 5 females. For both groups, measurements of pulmonary function were made under the same setting on two consecutive days during one week without exposure to cooking fumes, and then on the same weekdays during one subsequent week with exposure in an experimental setting.

The subjects were exposed to controlled levels of cooking fumes during the pan-frying of beef in a model kitchen of 56 m^3 ^(2.5 × 4 × 5.6 m) by use of an electric hob for group A and a gas hob for group B. The door and the window were kept closed, and the only ventilation was a kitchen ventilator which exhaled air at a rate of up to 600 m^3^/h. The level of cooking fumes in the kitchen was regulated by adjusting the quantity of beef in the pan, the extraction rate of the kitchen ventilator, and the effect level of the hotplate or the gas burner. The concentration of cooking fumes was monitored with a MIE pDR-1200 optical aerosol monitor (Thermo Andersen Inc., Smyrna, USA) located on a table 1.5 m from the cooking device and set to register the concentration of PM5 aerosols. The level was kept between 8–10 mg/m^3 ^for group A, and 10–14 mg/m^3 ^for group B. Group A was exposed to cooking fumes in the kitchen for 2 hours, with each person performing the frying 3 times for approximately 15 minutes each time, while group B was exposed for 4 hours with each person frying 3 times for approximately 25 minutes each time.

The sampling of total particles was performed using pre-weighed, double Gelman AE glassfiber filters (37 mm). The filters were placed in a closed face, clear styrene, acrylonitrile (SAN) cassette connected to a pump (Casella Vortex standard 2 personal air sampling pump, Casella CEL, Bedford, England) with an air flow of 2 l/min. The filters were placed on the right shoulder of the participant. Before and after sampling, the filters were conditioned in an exicator for 24 hours. The filters were analyzed gravimetrically, using a Mettler weight (0.01 mg dissolution). An inner calibration was performed on the weight before every weighing. Blank filters were included in the analysis in order to control for deviations caused by temperature or humidity.

The pulmonary function of the participants was measured with standard spirometry (Spirare sensor model SPS 310 based on tachopneumographic principles) and data were registered and analysed by the Spirare 3 software (Diagnostica corp., Norway). Spirometric parameters were measured with the subject in a sitting position, wearing a nose-clip, and breathing through the mouthpiece. Standardised instructions were given according to the criteria of American Thoracic Society[32]. We measured forced vital capacity (FVC), forced expiratory volume in one second (FEV1), peak expiratory flow (PEF), forced expiratory flows at 25, 50, and 75% of the vital capacity (FEF25, FEF50, FEF75), and forced expiratory time (FET), defined as the time from the start of the expiratory manoeuvre until the beginning of the end-expiratory plateau. The values used in the analysis were from the best curve out of three qualified performances. The best measurement was defined as that with the greatest sum of FEV1 and FVC. Measurements were done at four occasions for each person both during the week without exposure ("blind") and during the week with exposure to cooking fumes: 1) in the morning before entering the kitchen (between 8 and 9 am), 2) when leaving the kitchen after two hours (between 10 and 11 am (group A)), or four hours (between 12 am and 1 pm (group B)), 3) six hours after entering the kitchen (between 2 and 3 pm), and 4) twenty-four hours after entering the kitchen (between 8 and 9 am). The programme on the "blind day" was exactly the same as on the day with exposure in regard to location and activities, except that the subjects did not fry any beef, and were not exposed to any cooking fumes. In this way, the subjects were their own controls, making it possible to compare each subject's change in pulmonary function on a day with short term exposure to cooking fumes, with the change in pulmonary function on a day without exposure. Predicted values were based on a European reference material [33].

Results were registered and analyzed using SPSS for Windows version 14. Spearmen-Rank test was used to compare the intra-individual change in pulmonary function during the day with exposure, to the intra-individual change during the day without exposure. A significance level of 5% was chosen, and all statistical test results were two-sided.

The study was approved by the ethical committee for medical research in Central Norway. The participation was entirely voluntary, and written information was given to every participant about the project, also stating that he/she at any time could withdraw from the study. All participants received a symbolic allowance for their participation. There were no known conflicts of interest for any of the authors.

## Results

**Figure 1:**
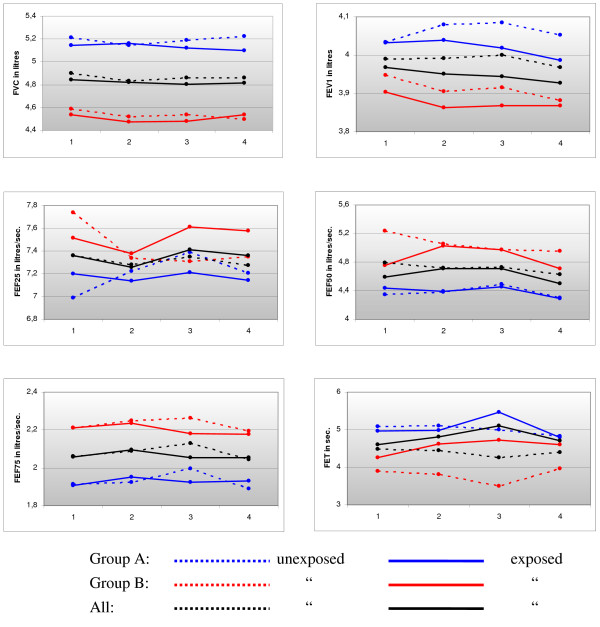
**Development of selected spirometric varaiables from 1) Just before entering the model kitchen, 2) When leaving it after 2 (group A) or 4 (group B) hours, 3) Six hours after entering, and 4) 24 hours after entering (next morning)**.

Table 1 shows the individual levels of exposure to cooking fumes, and some background variables for group A (participants 1–12) and group B (participants 13–24). The individual level of exposure measured by gravimetric analysis ranged from 13.8 to 32.9 mg/m^3 ^for group A, and from 31.2 to 54.9 mg/m^3 ^for group B. The mean spirometric performance of the participants on the first unexposed morning and the mean percent of their predicted values are shown in Table 2. Group A had a higher mean forced vital capacity (FVC) and forced expiratory volume in one second (FEV1), but the groups have about the same results relative to the percent of predicted values. Table 3 shows the changes in spirometric performance during the course of the days with and without exposure, while figure 1 shows the courses of some selected spirometric values as such.

**Table 1:** Personal exposure to particles from cooking fumes and personal characteristics of the twenty-four volunteers who participated in the study.

Group and subject number		Personal Exposure mg/m3	Sex*	Age (Years)	Height (cm)	Weight (Kg)	Current cold	Known allergy	Current medication
A	1	13.8	F	24	173	65	No	No	No
	2	14.4	M	25	193	105	No	No	No
	3	14.9	F	24	163	61	No	No	Yes
	4	13.9	F	21	152	45	No	No	No
	5	18.9	M	24	183	90	Yes	No	No
	6	20.8	F	22	166	66	No	Yes	Yes
	7	24.1	M	26	193	95	No	No	No
	8	24.4	M	28	177	75	No	No	No
	9	15.4	M	26	184	76	No	Yes	No
	10	32.9	M	25	172	67	Yes	No	No
	11	23.7	M	25	187	74	No	No	No
	12	16.7	M	24	187	84	Yes	Yes	No

All group A mean (SD)		19,5 (5,9)	50% female	24.5 (1.8)	177.5 (12.7)	75.3 (16.4)	25%	25%	17%

B									
	13	33.1	F	24	172	65	No	No	Yes
	14	43.2	M	23	185	73	Yes	Yes	No
	15	50.1	F	21	165	65	Yes	No	No
	16	32.8	F	21	162	49	No	Yes	Yes
	17	53.2	F	24	166	85	No	No	Yes
	18	31.9	M	23	187	86	No	Yes	No
	19	38.6	F	19	170	58	No	No	No
	20	31.2	M	31	176	78	No	No	No
	21	52.5	M	21	165	68	No	Yes	Yes
	22	44.8	M	25	171	63	No	No	No
	23	47.3	M	22	169	63	Yes	Yes	Yes
	24	54.9	M	23	180	95	No	No	Yes

All group B mean (SD)		42,8 (9,0)	42% female	23.1 (3.0)	172.3 (8.1)	70.7 (13.2)	25%	42%	50%

All 24 mean (SD)		31,1 (14,0)	46% female	23.8 (2.5)	174.9 (10.8)	73.0 (14.7)	25%	33%	33%

F = female, m = male

**Table 2:** Spirometric values measured in the two groups and % of predicted values.

Spirometric measure	Group A		Group B		All	
			
	Mean (SD)	% of pred	Mean (SD)	% of pred	Mean (SD)	% of pred.
FVC, litres	5.2 (1.3)	105	4.6 (1.0)	101	4.9 (1.2)	103
FEV1, litres	4.0 (0.8)	95	3.9 (0.8)	102	4.0 (0.8)	99
FEV%	79.0 (8.8)	n.a.	86.2 (3.6)	n.a	82.6 (7.5)	n.a.
PEF litres/min	570 (112)	103	550 (126)	106	560 (117)	104
FEF25 litres/sec.	7.0 (1.6)	88	7.7 (1.7)	104	7.4 (1.7)	96
FEF50 litres/sec	4.3 (0.8)	78	5.2 (1.1)	102	4.8 (1.0)	91
FEF75 litres/sec	1.9 (0.3)	73	2.2 (0.5)	92	2.1 (0.5)	84
FET seconds	5.1 (1.1)	n.a.	3.9 (1.2)	n.a.	4.5 (1.3)	n.a.

n.a. = not applicable

**Table 3:** Percentual changes in spirometric values at different points in time in the groups and during periods with (E) and without (B) exposure to cooking fumes.

Spirometric measure		Group A (n = 12)			Group B (n = 12)			All (n = 24)		
				
		2-1#	3-1	4-1	2-1	3-1	4-1	2-1	3-1	4-1
FVC	B	-1.1	-0.6	+0.1	-1.7	-1.3	-2.3	-1.4	-0.9	-1.1
	E	+0.2	-0.5	-0.8	-1.3	-0.9	+0.1	-0.6	-0.7	-0.4
										
FEV1	B	+1.1	+1.3	+0.6	-0.8	-0.6	-1.6	+0.2	+0.4	-0.5
	E	+0.5	-0.5	-1.2	-0.8	-0.5	-0.5	-0.2	-0.5	-0.9
										
FEV%	B	+2.3	+1.9	+0.6	+0.9	+0.8	+0.7	+1.6	+1.4	+0.7
	E	+0.3	+0.0	-0.3	+0.5	+0.5	-0.6	+0.4	+0.2	-0.5
										
PEF	B	+2.4	-0.5	-1.7	-1.7	-1.7	-3.0	+0.4	-1.1	-2.3
	E	-0.8	-0.2	-0.6	+0.9	+2.6	+1.4	+0.1	+1.2	+0.4
										
FEF25	B	+3.8	+5.9	+3.6	-5.0	-5.4	-4.2	-0.6	+0.3	-0.3
	E	-0.9	+0.5	-0.4	-1.4	+1.9*	+0.7	-1.2	+1.2	+0.1
										
FEF50	B	+0.6	+3.4	-0.2	-2.6	-4.5	-4.6	-1.0	-0.6	-2.4
	E	-0.6	+0.7	-2.5	+6.5*	+6.1*	+0.6	+2.9	+3.4	-1.0
										
FEF75	B	-0.7	+3.7	-0.9	+3.8	+3.8	+0.1	+1.6	+3.7	-0.4
	E	+2.3	+0.6	+0.9	+1.3	-1.0	-0.6	+1.8	-0.2	+0.1
										
FET	B	+1.0	+0.2	-4.5	-0.7	-6.7	+8.7	+0.1	-3.2	+2.1
	E	+1.0	+16.9	+1.0	+12.8	+14.6	+7.3	+6.9	+15.7*	+4.2

* p < 0.05

# 2-1 is the difference between the first measurement and the measurement at the time of leaving the kitchen after 2 or 4 hours. 3-1 is the difference between the first measurement and the measurement taken 6 hours after entering the kitchen. 4-1 is the difference between the first measurement and the measurement taken 24 hours after entering the kitchen during the day with exposure compared to the day without exposure.

The forced expiratory time (FET) on entering the kitchen compared to the FET six hours later was significantly altered, with a 15.7% increase on the exposed day, compared to a 3.2% decrease during the "blind day" (p-value = 0.03).

The same tendency can be seen for FET measurements done immediately after the exposure and on the next morning, but this was not statistically significant. For the forced expiratory flow when 50% is exhaled (FEF50), group B showed a statistically significant increase between both the first and the second (2-1) and the first and the third (3-1) measurements.

For FEF25 (when 25% is exhaled), a similar difference was found between the first and the third measurement (3-1). We found no statistically significant differences between the changes in other spirometric measurements during the day of exposure, compared to the changes during the "blind day".

## Discussion

Most previous studies of effects from cooking fumes have looked at manifest diseases and chronic respiratory effects in cooks and other exposed groups[14-18,26-29]. In this study we aimed to determine early, short term changes in lung function in healthy subjects subsequent to exposure to cooking fumes in an experimental setting. In such a setting we did not expect to find dramatic changes in crude spirometric measures such as FVC, FEV1 or PEF, but rather hypothesised that there might be changes in measures that reflected more the function of the small airways, such as FEF 75 and FET.

In our paired analysis it was shown that FET developed differently during the day of exposure, compared to the "blind day". Prolonged FET has been associated with obstructive disorders[34], and abnormalities in FET have been found in symptomatic smokers with normal FEV1[35]. FET has been suggested as a measure of small airways obstruction[36]. It has been found to have an important discriminatory ability[37], but also a rather low repeatability[37-39]. A recent population study found that FET had a high coefficient of variation (CoV) of 11.3% compared to FVC, FEV1, and PEF which had CoV of 1.38%, 1.44% and 3.0% respectively [38]. It has also been shown that airflow limitation tends to prolong FET, even in healthy subjects [40]. The increase in FET during the day of exposure in our study might thus be explained by inflammatory responses and an obstruction in the distant peribronchiolar tissue caused by the inhalation of cooking fumes. It has, however, been claimed that there is an association between improved spirometric performance and the FET, and that repeated measurements can lead to a training effect[41]. The increase in FET during the day of exposure, which was subsequent to the "blind day", could therefore alternatively be explained by better spirometric performance resulting from a training effect. However, if a learning response was the explanation for the prolonged FET in our study, one would expect to have an increase in FET during the blind day as well, but instead, a decrement in FET appeared. Moreover, if a prolonged FET should be seen as a result of a training effect, the change would probably have gone along with an increase in the FVC and other parameters as well. The lack of such an improvement in our study makes the possibility of a learning effect in regard to the observed increase in FET less probable, in our view.

Although the other spirometric parameters did not develop significantly differently on the "blind" day and the day with exposure, there might have been a tendency. We find it interesting that the mean FEV1 increased by 0.4% from the morning until 2 – 3 pm on the "blind" day, while it decreased by 0.5% during the same period of time on the day with exposure (Table 3 and Figure 1). The increase of FEV1 during the blind day could reflect diurnal variation. In a recent study FEV1 in young adults was shown to increase by 120 ml from 9.00 A.M. until noon, and decreased a little in the afternoon[42]. The diurnal variation of FEV1 was, however, shown to be less pronounced in those who were without symptoms and non-smokers. As our subjects were young, a certain increase in FEV1 from the morning till noon could be expected. On the other hand, all of our subjects were both symptom-free and non-smokers, which might explain the low observed diurnal variation of FEV1 in our study. Also, in the statistical analysis the diurnal variation was controlled for since the change in spirometry was compared between weeks with measurements at the same points of time. The observation of some statistical improvement in FEF25 and FEF50 in group B on the day with exposure compared to the day without was unexpected. When exploring the data, three subjects from group B had unusual, and unexplainably high, starting values for these variables solely on the day without exposure (point 1, dotted line in figure 1). Thus, the difference could as much be due to an unexplainable fall in these measurements on the blind day as due to the slight increase on the exposed day. When the three subjects with the unusual starting values were taken out of the analysis, there were no statistically significant differences.

One possible interpretation of the lack of statistically significant changes in other spirometric measures than the FET could be that the twenty-four subjects that we had access to might be too few to render enough statistical power when studying small changes in the airways. Thus, we cannot conclude that some other parameters of the pulmonary function were not affected, even though we could not detect any significant differences between the "blind" day, and the exposed day.

We think that the chosen short term exposure of the groups to cooking fumes was quite realistic. Both for group A and B, the exposure was at a level that led to subjective annoyance; thus we did not find it right to make it any higher. Even so, it might still have been too low in both groups to irritate the lungs enough to give a short term response that can be measured by more spirometric parameters. By gravimetrical analyses of the personal filters carried by the participants, the exposure seemed to be higher than the levels measured on a stationary basis by the MIE instrument in the model kitchen. The reason for this was most likely that the MIE instrument was placed 1.5 meters away from the hob, while the filters were mounted near the breathing zone of the subjects, and thus came closer to the hob when the subjects were actually frying beef.

With regard to the duration of the exposure, both two hours (group A) and four hours (group B) might have been too short to give a short term response that can be measured by more spirometric parameters. On the other hand, other studies have been able to unveil spirometric changes over relatively short time spans[30,31]. It should also be recognised that there were no differences in changes in lung function between group A and B, even though group B had a mean cumulative exposure (degree × time) that was more than four times as high as for group A. Thus, the study did not unveil any relationship between cumulative exposure and lung function changes. One should also be aware that there were other differences in exposure between the groups in that group A worked with an electrical hob, while B had a gas hob without observed differences in spirometric changes.

## Conclusion

In conclusion, there seems, in our experimental setting, to be minor short term spirometric effects from exposure to cooking fumes, mainly affecting FET.

## Competing interests

The authors declare that they have no competing interests.

## Authors' contributions

SS participated in the design of the study, drafting the manuscript and in performing the statistical analyses. BH participated in the design of the study, drafting the manuscript and in performing the statistical analyses. TQ participated in the design of the study. AKS contributed to the manuscript and was responsible for the exposure conditions. KS participated in the design of the study, contributed to the manuscript and in performing the statistical analyses. All authors participated during the execution of the experimental. All authors read and approved the final manuscript.

## References

[B1] Kiel P (1986). Kræft og stegeos. (Cancer and cooking fumes). Research reports from the Danish Working Environment Research Fund Copenhagen.

[B2] Kiel P, Andersen M (1988). Mutagener i stegeos. (Mutgenic substances in cooking fumes). Research reports from the Danish Working Environment Reasearch Fund Copenhagen.

[B3] Vainiotalo S, Matveinen K (1993). Cooking fumes as a hygienic problem in the food and catering industries. Am Ind Assoc J.

[B4] Robinson AL, Subramanian R, Donahue NM, Bernardo-Bricker A, Rogge WF (2006). Source apportionment of molecular markers and organic aerosol. 3. Food cooking emissions. Environ Sci Technol.

[B5] Ghilarducci DP, Tjeerdema RS (1995). Fate and effects of acrolein. Rev Environ Contam Toxicol.

[B6] Ross J, Seaton A, Morgan W, Morgan W, Seaton A (1995). Toxic gases and fumes. Occupational lung diseases.

[B7] Costa DL, Klaassen CD (2008). Air pollution. Casarett and Doull's Toxicology: the basic science of poisons.

[B8] Jensen LK, Larsen A, Molhave L, Hansen MK, Knudsen B (2001). Health evaluation of volatile organic compound (VOC) emissions from wood and wood-based materials. Arch Environ Health.

[B9] Li S, Pan D, Wang G (1994). Analysis of polycyclic aromatic hydrocarbons in cooking oil fumes. Arch Environ Health.

[B10] Thiebaud HP, Knize MG, Kuzmicky PA, Hsieh DP, Felton JS (1995). Airborne mutagens produced by frying beef, pork and a soy-based food. Food Chem Toxicol.

[B11] Mingzhen C, Zhenyang C, Zhenhua Z (1995). Benzo[a]pyrene in Kitchen Air and Urinary 1-Hydroxypyrene. Indoor Built Environt.

[B12] Yang CC, Jenq SN, Lee H (1998). Characterization of the carcinogen 2-amino-3,8-dimethylimidazo[4,5-f]quinoxaline in cooking aerosols under domestic conditions. Carcinogenesis.

[B13] Chiang T-A, Wu P-F, Ko Y-C (1999). Identification of Carcinogens in Cooking Oil Fumes. Environ Res.

[B14] Coggon D, Pannett B, Osmond C, Acheson ED (1986). A survey of cancer and occupation in young and middle aged men. I. Cancers of the respiratory tract. Br J Ind Med.

[B15] Lund E (1986). Kokker og dødelighet av kreft. (Cancer and mortality in cooks). Rapport fra Direktoratet for Arbeidstilsynet Oslo: Direktoratet for Arbeidstilsynet.

[B16] Zhong L, Goldberg MS, Parent ME, Hanley JA (1999). Risk of developing lung cancer in relation to exposure to fumes from Chinese-style cooking. Scand J Work Environ Health.

[B17] Yang SC, Jenq SN, Kang ZC, Lee H (2000). Identification of benzo[a]pyrene 7,8-diol 9,10-epoxide N2-deoxyguanosine in human lung adenocarcinoma cells exposed to cooking oil fumes from frying fish under domestic conditions. Chem Res Toxicol.

[B18] Zhou BS, Wang TJ, Guan P, Wu JM (2000). Indoor air pollution and pulmonary adenocarcinoma among females: a case-control study in Shenyang, China. Oncol Rep.

[B19] International Agency for Research on Cancer (2006). Monographs on the Evaluation of Carcinogenic Risks to Humans Indoor air pollution from household cooking and heating: Emissions from high-temperature frying.

[B20] Oldenburger D, Maurer WJ, Beltaos E, Magnin GE (1972). Inhalation lipoid pneumonia from burning fats. A newly recognized industrial hazard. Jama.

[B21] Kennedy JD, Costello P, Balikian JP, Herman PG (1981). Exogenous lipoid pneumonia. AJR Am J Roentgenol.

[B22] Spickard A, Hirschmann JV (1994). Exogenous lipoid pneumonia. Arch Intern Med.

[B23] Robertson AS, Weir DC, Burge PS (1988). Occupational asthma due to oil mists. Thorax.

[B24] Eisen EA, Tolbert PE, Monson RR, Smith TJ (1992). Mortality studies of machining fluid exposure in the automobile industry I: A standardized mortality ratio analysis. Am J Ind Med.

[B25] Kazerouni N, Thomas TL, Petralia SA, Hayes RB (2000). Mortality among workers exposed to cutting oil mist: Update of previous reports. Am J Ind Med.

[B26] Ng TP, Tan WC (1994). Epidemiology of allergic rhinitis and its associated risk factors in Singapore. Int J Epidemiol.

[B27] Ng TP, Hui KP, Tan WC (1993). Respiratory symptoms and lung function effects of domestic exposure to tobacco smoke and cooking by gas in non-smoking women in Singapore. J Epidemiol Community Health.

[B28] Svendsen K, Sjaastad AK, Sivertsen I (2003). Respiratory symptoms in kitchen workers. Am J Ind Med.

[B29] Borgan J, Kristoffersen L (1986). Dødelighet i yrker og sosioøkonomiske grupper 1970–1980. (Mortality by occupation and socio-economic group in Norway 1970–1980) Statistiske Analyser, No. 56.

[B30] Bakke B, Ulvestad B, Stewart P, Lund MB, Eduard W (2001). Effects of blasting fumes on exposure and short-term lung function changes in tunnel construction workers. Scand J Work Environ Health.

[B31] Skogstad M, Kjaerheim K, Fladseth G, Gjolstad M, Daae HL, Olsen R, Molander P, Ellingsen DG (2006). Cross shift changes in lung function among bar and restaurant workers before and after implementation of a smoking ban. Occup Environ Med.

[B32] American Thoracic Society (1995). Standardization of Spirometry: 1994 update. Am J Respir Crit Care Med.

[B33] Quanjer PH, Tammeling GJ, Cotes JE, Pedersen OF, Peslin R, Yernault JC (1993). Lung volumes and forced ventilatory flows. Report Working Party Standardization of Lung Function Tests, European Community for Steel and Coal. Official Statement of the European Respiratory Society. Eur Respir J Suppl.

[B34] Lal S, Ferguson AD, Campbell EJ (1964). Forced Expiratory Time: A Simple Test for Airways Obstruction. Br Med J.

[B35] McFadden ER, Linden DA (1972). A reduction in maximum mid-expiratory flow rate. A spirographic manifestation of small airway disease. Am J Med.

[B36] Cochrane GM, Benatar SR, Davis J, Collins JV, Clark TJ (1974). Correlation between tests of small airway function. Thorax.

[B37] Miller MR, Pincock AC (1982). Repeatability of the moments of the truncated forced expiratory spirogram. Thorax.

[B38] Kainu A, Lindqvist A, Sarna S, Sovijarvi A (2008). Intra-session repeatability of FET and FEV6 in the general population. Clin Physiol Funct Imaging.

[B39] Cochrane GM, Prieto F, Clark TJ (1977). Intrasubject variability of maximal expiratory flow volume curve. Thorax.

[B40] Kainu A, Lindqvist A, Sarna S, Sovijarvi A (2008). Spirometric and anthropometric determinants of forced expiratory time in a general population. Clin Physiol Funct Imaging.

[B41] Tsai AG, Christie JD, Gaughan CA, Palma WR, Margolis ML (2006). Change in forced expiratory time and spirometric performance during a single pulmonary function testing session. Respir Care.

[B42] Borsboom GJ, van Pelt W, van Houwelingen HC, van Vianen BG, Schouten JP, Quanjer PH (1999). Diurnal variation in lung function in subgroups from two Dutch populations: consequences for longitudinal analysis. Am J Respir Crit Care Med.

